# Construction of supramolecular nanotubes from protein crystals†
†Electronic supplementary information (ESI) available. See DOI: 10.1039/c8sc04167a


**DOI:** 10.1039/c8sc04167a

**Published:** 2018-10-30

**Authors:** Tien Khanh Nguyen, Hashiru Negishi, Satoshi Abe, Takafumi Ueno

**Affiliations:** a School of Life Science and Technology , Tokyo Institute of Technology , Nagatsuta-cho , Midori-ku , Yokohama 226-8501 , Japan . Email: tueno@bio.titech.ac.jp

## Abstract

Investigations involving the design of protein assemblies for the development of biomaterials are receiving significant attention. In nature, proteins can be driven into assemblies frequently by various non-covalent interactions. Assembly of proteins into supramolecules can be conducted under limited conditions in solution. These factors force the assembly process into an equilibrium state with low stability. Here, we report a new method for preparing assemblies using protein crystals as non-equilibrium molecular scaffolds. Protein crystals provide an ideal environment with a highly ordered packing of subunits in which the supramolecular assembled structures are formed in the crystalline matrix. Based on this feature, we demonstrate the self-assembly of supramolecular nanotubes constructed from protein crystals triggered by co-oxidation with cross-linkers. The assembly of tubes is driven by the formation of disulfide bonds to retain the intermolecular interactions within each assembly in the crystalline matrix after dissolution of the crystals.

## Abstract 2

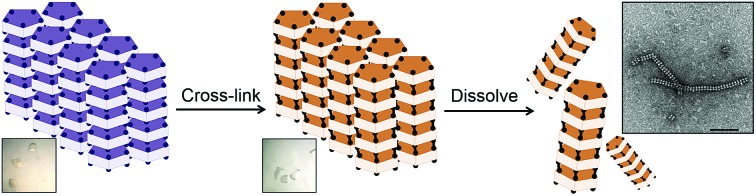
Cross-linking of protein crystals promotes disulfide-mediated nanotubes.

## Introduction

Recently, engineering of supramolecular protein assemblies has been demonstrated as a useful strategy for generating materials with periodical architectures having rational design,^1^–^4^ ease of control,^5^–^8^ and built-in reactivity.^9^–^12^ Construction of artificial protein assemblies is useful for the development of artificial enzymes,^13^,^14^ containers of molecules,^15^–^18^ reaction vessels,^19^–^22^ and targeted therapeutics^23^–^25^ because proteins can self-assemble into sophisticated topologies, such as cages,^26^ tubes,^27^ and sheets^28^ which can be used as nanomaterials in various biological applications.^29^ Although these assemblies can be retained *via* metal-coordination, charge–charge interactions, and covalent bonds,^30^,^31^ it remains challenging to construct such artificial assemblies from aqueous solutions of proteins due to difficulties in controlling protein dynamics and protein–protein interactions under equilibrium conditions, which depends on the interference of various factors, such as pH, ionic strength, temperature, and the presence of various co-solutes.^30^

Protein crystals are promising candidates for use in designing architectures of supramolecules because the highly ordered arrangements of a crystalline lattice provide an environment, which enables the formation of a protein self-assembly that is not efficiently formed in aqueous solution.^32^ By crystallization, we can transform the protein–protein interactions from non-specific interactions to specific interactions, and from an equilibrium state to a non-equilibrium state, compared to proteins in solution. Indeed, it has been demonstrated that engineering of protein crystals generates varieties of integrated materials, such as organometallic composites,^33^,^34^ photoactive protein crystals,^35^,^36^ polymer conjugated crystals,^37^,^38^ and artificial assemblies.^39^ Based on their highly ordered structures, it is expected that protein crystals will provide useful materials for the construction of supramolecular assemblies with desired architectures.

To functionalize and stabilize the protein crystals, several methods have been developed.^40^,^41^ Disulfide bonds that form between the thiol groups of closed cysteine (Cys) residues within the crystals *via* oxidation are demonstrated to enhance the stability of the assembly within the crystalline environment.^39^,^42^,^43^ The stable assembly of *in vivo* polyhedral crystals allows the release of the caged structure after dissolution in a buffer solution.^39^ However, the oxidation of cysteine sites within protein crystals provides a significant constraint of distances that cannot be altered efficiently. It was recognized that utilization of cross-linkers could address this problem and provide additional control in stabilizing an assembly in a protein crystal as reported for the construction of a protein assembly in solution.^32^,^44^ After cross-linking, the crystals remain stable under harsh conditions, such as high temperature and the presence of organic solvents.^32^,^40^ On the other hand, cross-linking of thiols can induce the formation of periodical assembled nanostructures with nanotube and sheet structures.^1^,^44^–^46^ Thus, it is expected that the precise placement of cysteines at specific sites will permit cross-linking to occur within the crystalline environment to stabilize the assembly with the structure of interest that can be released after dissolution of crystals in aqueous solution.

**Fig. 1:**
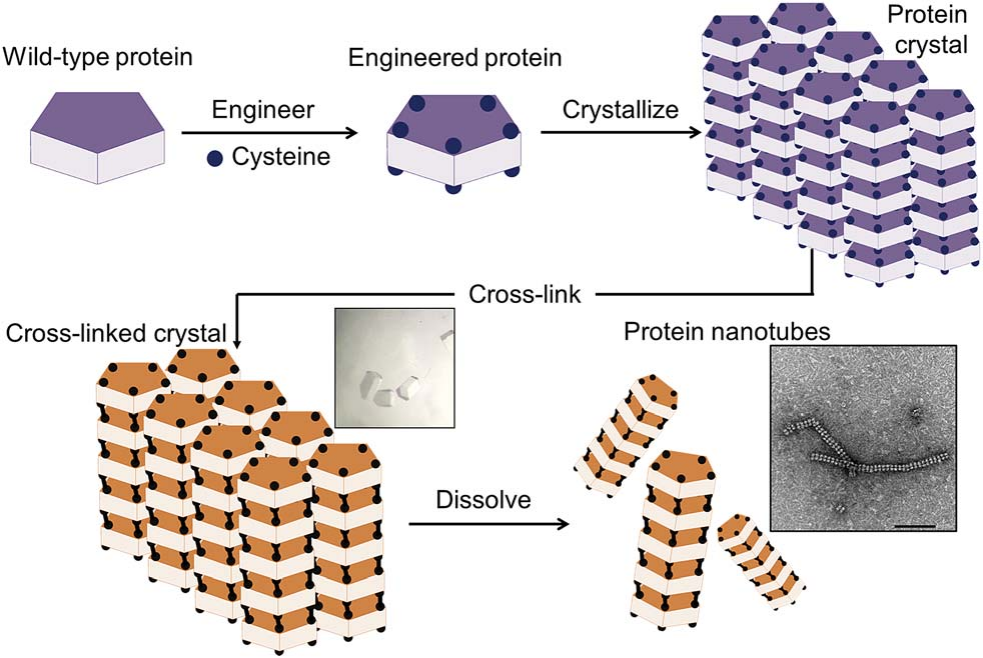
Schematic representation of the construction of supramolecular nanotubes from protein crystals by introduction of cysteine residues into the wild-type protein at the interface, crystallization of the engineered protein into a typical lattice, cross-linking of the protein crystal, and dissolution.

Here, we demonstrate a strategy for the construction of supramolecular nanotubes in protein crystals by (1) design of protein interfaces with introduction of Cys sites, (2) crystallization of the engineered protein to achieve the crystalline assembly as expected, (3) initiating a cross-linking reaction in the engineered crystal to induce the formation of intermolecular disulfide bonds under oxidation conditions triggered by hydrogen peroxide (H_2_O_2_) in the presence and absence of cross-linkers, and (4) dissolution of the cross-linked crystal in buffer solution to release the desired assembled structures (Fig. 1). In this strategy, the crystalline matrix can provide a convenient environment for site-specific cross-linking to enable the formation of nanotubes that cannot be achieved in aqueous solutions of proteins. Furthermore, this strategy represents an advanced method for controlling the construction of protein nanotubes under non-equilibrium conditions. The length of the nanotubes can be controlled under different cross-linking conditions of engineered crystals with the linkers, while the protein in solution or wild-type crystal would be restricted from forming nanotubes under the same conditions. Here, we report the engineering of a three-dimensional protein crystal for the construction of tubular structures induced by co-oxidation with cross-linkers.

## Results

### Design of the cross-linked cysteine sites in the protein crystal

**Fig. 2:**
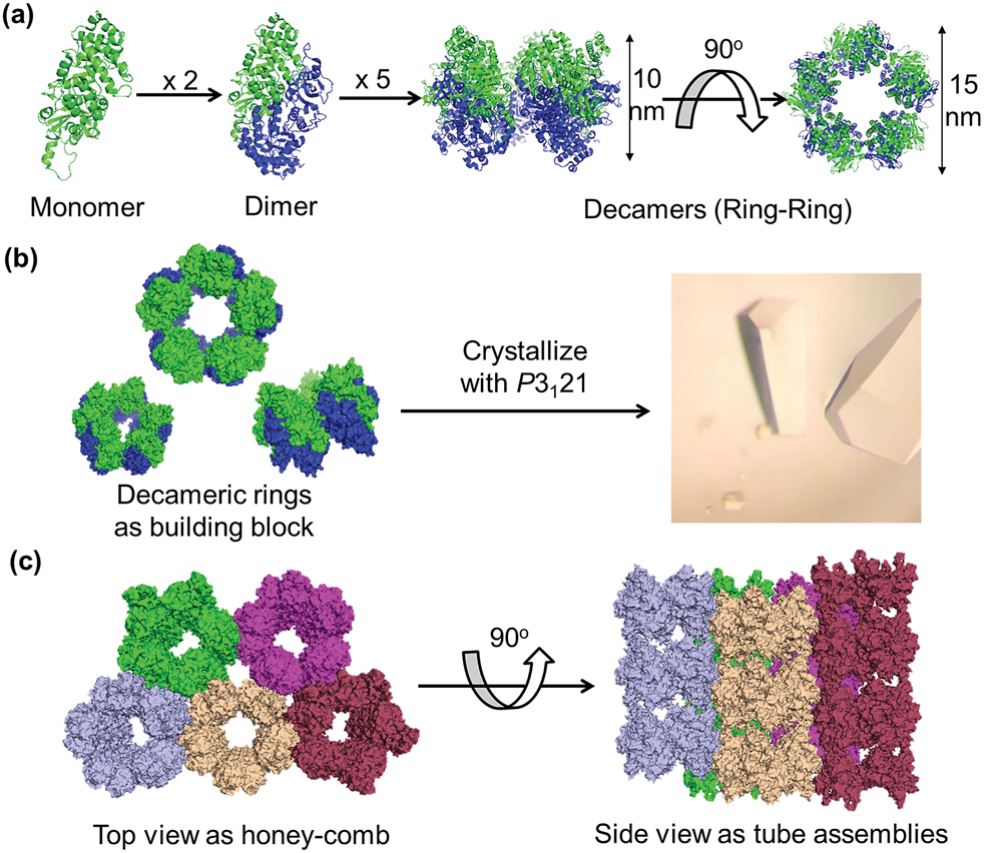
(a) Natural assembly of RubisCO as a protein building block. (b) Crystallization of RubisCO with the *P*3_1_21 space group. (c) Crystal lattice structure of the space group *P*3_1_21 in top and side views.

**Fig. 3:**
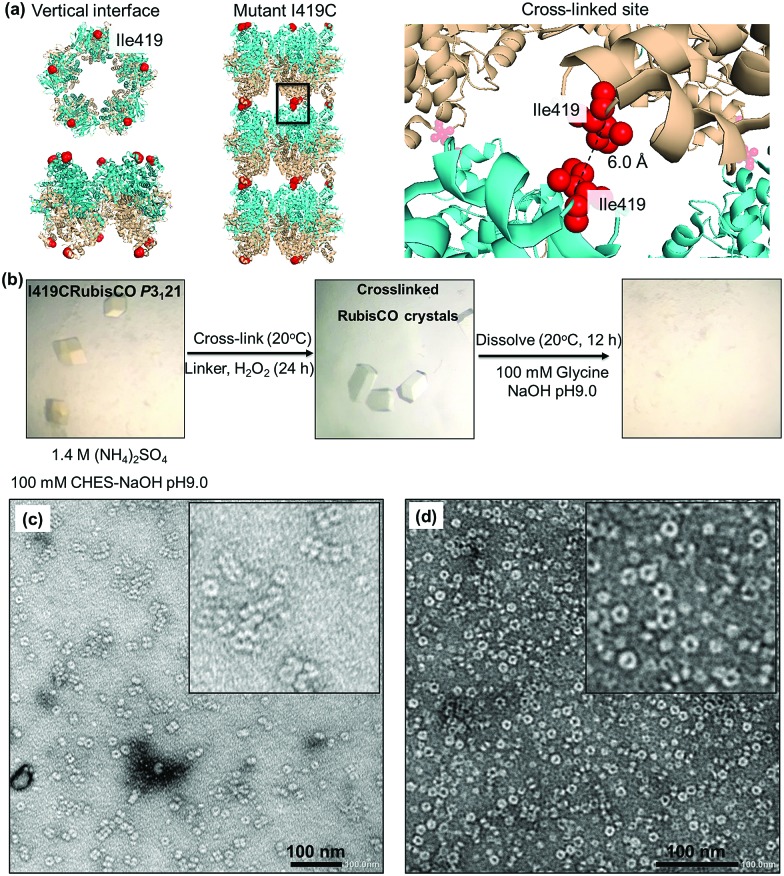
(a) Engineering of the WTRubisCO crystal of lattice *P*3_1_21 with the mutant I419CRubisCO. (b) Experimental procedure for the construction of supramolecular nanotubes by cross-linking and dissolution. (c) Protein nanotubes obtained from the oxidation of I419CRubisCO crystals with 1 mM H_2_O_2_. (d) Assembly ring of RubisCO obtained from the dissolution of I419CRubisCO crystals without oxidation.

Ribulose-1,5-bisphosphate carboxylase/oxygenase type III from the archaeon *Thermococcus kodakaraensis* KOD1 (RubisCO) was employed as a protein building block. RubisCO is a natural decameric assembly composed of 10 identical subunits stabilized by non-covalent interactions, forming a stacking system of double-pentameric rings with *C*_5_ symmetry (Fig. 2a). For the construction of a tubular assembly from the crystal, RubisCO has three advantages: (1) it has high stability towards retention of the double five-membered ring structure, (2) the wild-type RubisCO (WTRubisCO) is a free-Cys protein, and (3) the arrangement of the crystal with the space group of *P*3_1_21 includes a 1D tubular assembly structure (Fig. 2b, c).^47^,^48^ It is expected that introduction of Cys at specific sites in the *P*3_1_21 lattice will facilitate the formation of intermolecular disulfide bonds at the interfaces of each decameric assembly. We focused on the decameric ring–ring interfaces along the *C*_5_ symmetry axis to identify a position for the introduction of Cys residues. Ten isoleucine 419 (Ile419) residues are located at two exterior surfaces along the *C*_5_ symmetry (Fig. 3a). At each surface, five Ile419 residues are located at the face-to-face position with adjacent five Ile residues of the neighboring decameric ring with a C_α_–C_α_ distance of 6 Å (Fig. 3a). Therefore, we designed the mutant I419CRubisCO because a C_α_–C_α_ distance of 6.5 Å between Cys residues is expected to be a feasible distance for the formation of a disulfide bond with oxidation promoted by H_2_O_2_.^49^,^50^

### Reaction of the I419CRubisCO crystal with H_2_O_2_

**Fig. 4:**
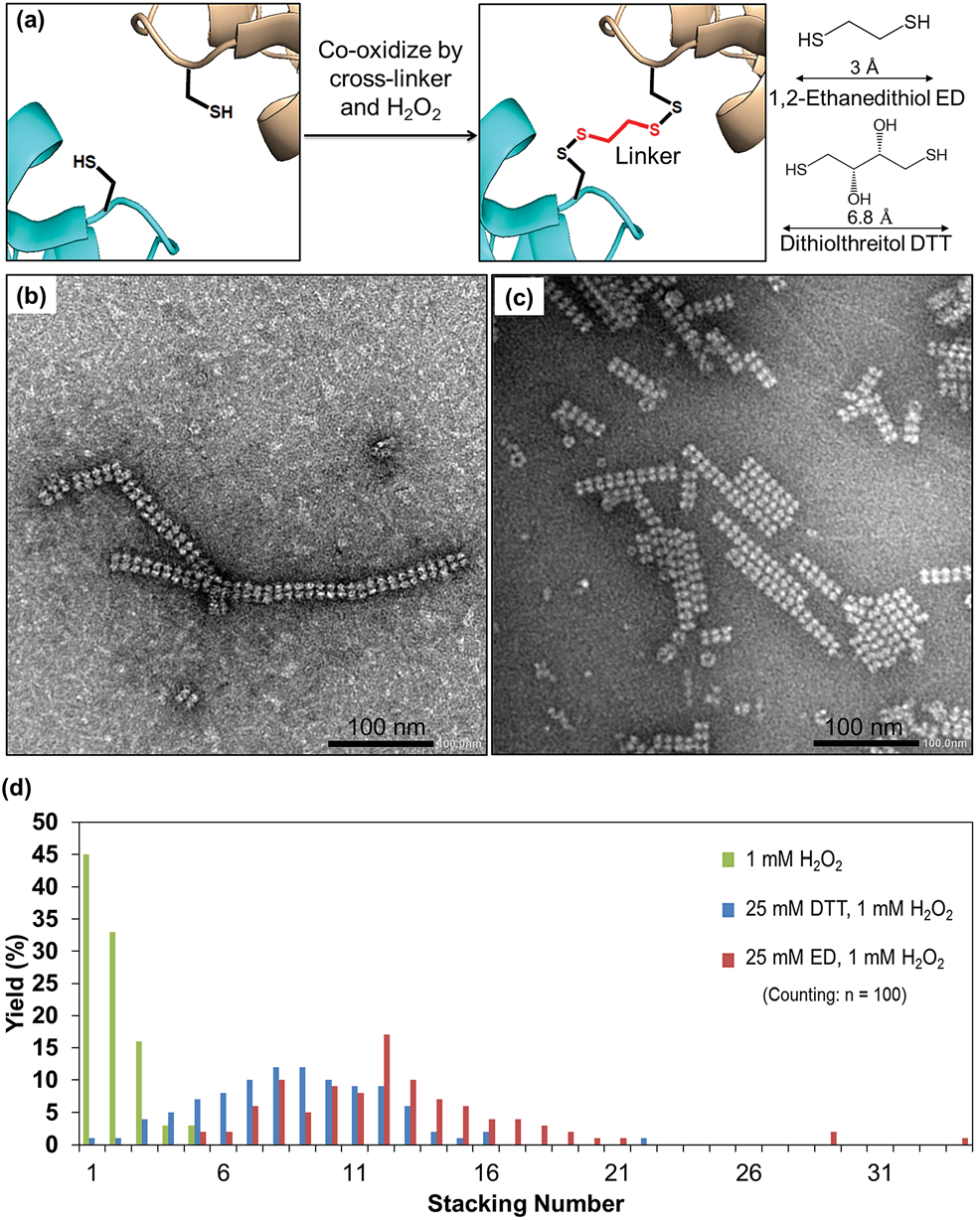
(a) Strategy of cross-linking based on the C_α_–C_α_ distance of cysteine in the presence of cross-linkers. (b) Protein nanotubes triggered by co-oxidation with 25 mM ED and 1 mM H_2_O_2_. (c) Protein nanotubes obtained from co-oxidation by 25 mM DTT and 1 mM H_2_O_2_. (d) Histogram indicating the effect of cross-linkers and types of cross-linkers on elongation of the protein nanotubes.

The mutant I419CRubisCO was crystallized under the same conditions as WTRubisCO as previously reported.^48^ The I419CRubisCO crystal retains the lattice structure which has an arrangement of bundles of tubular assemblies, as confirmed by transmission electron microscopy (TEM) (Fig. S1†). Although we could obtain the data from X-ray diffraction, we did not succeed in the analysis of the crystal structure because of the low resolution data. To prepare the nanotubes in the crystals, we first examined oxidation by using only H_2_O_2_. The I419CRubisCO crystals were soaked into a 1 mM H_2_O_2_ solution of the buffer used for crystallization (1.4 M (NH_4_)_2_SO_4_, 10 mM MgCl_2_, and 100 mM CHES–NaOH buffer (pH 9.0)) at 20 °C for 24 h to obtain the oxidized I419CRubisCO (Ox-I419CRubisCO) crystals (Fig. 3b). Then, the nanotubes were released by dissolution of Ox-I419CRubisCO crystals in 100 mM glycine–NaOH buffer (pH 9.0) at 20 °C for 12 h (Fig. 3b). The dissolved portion of the Ox-I419CRubisCO crystals was evaluated *via* TEM to confirm the formation of nanotubes. We observed mostly the non-cross-linked I419CRubisCO (Fig. 3c), with evidence being provided by the presence of mono-ring structures as natural decameric assemblies without oxidation treatment (Fig. 3d). In addition, very few nanotubes were identified with a maximum of 5 stacking units (approximately 60 nm in length compared to 12 nm length of natural decameric rings). This represents a yield below 5%, determined by counting the rings in the TEM images (Fig. 4d).

The results indicate that there is spatially confined formation of disulfide bonds between the I419C residues in the crystalline state. Although there is a C_α_–C_α_ distance of 6 Å between the adjacent Cys residues at position 419 in the crystalline lattice *P*3_1_21, the orientation or position of the sulfur atoms might not be suitable for the formation of disulfide bonds due to the restricted conformations of Cys side chains within the crystals. Thus, the nanotubes were generated in very low yield and with a few stacked layers. To overcome this issue, we recognized that cross-linkers can be employed in a co-oxidation reaction triggered by H_2_O_2_ to effectively promote site-specific cross-linking between Cys residues at position 419.

### Formation of protein nanotubes by co-oxidation of the crystal with cross-linkers

Cross-linking, a process of linking two different or identical proteins *via* linkers, has been demonstrated to stabilize protein–protein interactions.^51^ Cross-linking monomers within a protein crystal has significant potential for facilitating the preparation of inorganic nanoparticles, sensing materials, and drug carriers.^40^,^52^ The crystal can be cross-linked randomly with glutaraldehyde or precisely with targeted linkers at the thiols or amines of protein moieties.^32^,^53^ The highly ordered arrangements of protein molecules in the crystal offer a wide range of pore sizes and high porosity^32^,^54^ which allows the cross-linkers to access the cysteine sites in the crystalline matrix. The formation of nanotubes in the presence of different cross-linkers was evaluated. Since the distance between C_α_ atoms of the nearest cysteine is within 6.5 Å, it is expected that short cross-linker molecules are suitable to fit between the sulfur atoms. We chose two small cross-linking reagents, 1,2-ethanedithiol (ED) and dithiothreitol (DTT) (Fig. 4a). To prepare the co-oxidized nanotubes, the I419CRubisCO crystals were pre-soaked in 25 mM ED in the crystallization buffer (1.4 M (NH_4_)_2_SO_4_, 10 mM MgCl_2_, and 100 mM CHES–NaOH (pH 9.0)) at 20 °C for 8 h, followed by the oxidation process triggered by the addition of 1 mM H_2_O_2_ and an additional 16 h of incubation at 20 °C to obtain the co-oxidized I419CRubisCO crystals (CoOx-I419CRubisCO). The CoOx-I419CRubisCO crystals were dissolved in 100 mM glycine–NaOH (pH 9.0) at 20 °C for 12 h to release the nanotubes and then the solution was confirmed by TEM as shown in Fig. 4b.

ED promoted the elongation of nanotubes with lengths of about 400 nm and about 34 subunits (Fig. 4b, S2a†). The distribution of stacking numbers significantly shifts to higher layers of 11–13, which could not be provided by co-oxidation *via* 1 mM H_2_O_2_ without ED (Fig. 4d). Similar elongation of nanotubes was observed using DTT under the same co-oxidation conditions. DTT extends the nanotubes into the highest stacking units of 22 with an average distribution ranging from 8 to 10, making it less effective than ED (Fig. 4c, d, and S2b†). This observation could be explained by the effects of the different cross-linkers on the accessibility to the cysteine at position 419. The length of ED is 3 Å,^55^ which provides a more flexible structure to access the cross-linking sites which have a distance of 6 Å (C_α_–C_α_) between two thiol groups of adjacent Cys419 residues located face-to-face at the exterior surfaces. ED is more effective in the formation of disulfide bonds to elongate the tube structure than DTT, which has a longer length of 6.8 Å than ED.^56^

### Efficiency of the cross-linking reaction in a protein crystal

To confirm that the crystal plays vital roles in specific cross-linking to facilitate the release of nanotubes, we performed control experiments with oxidation of the I419CRubisCO solution by H_2_O_2_ in the presence and absence of cross-linkers. The results indicate that the solution of I419CRubisCO cannot form nanotubes under oxidized or co-oxidized conditions although all of the Cys residues were exposed on the exterior surfaces of the decameric rings. The assemblies were captured with a mixture of partially random cross-linkages as clusters of rings and short nanotubes with a maximum of 9 stacking units due to freely diffusing I419CRubisCO rings in the aqueous buffer solution (Fig. S2c–e†). These findings indicate that the crystalline environment is necessary to achieve the expected cross-linking sites to form the nanotubes. Indeed, the I419CRubisCO crystals exhibit arrangements of 1D tubular assemblies that enhance the formation of disulfide bonds along the *C*_5_ symmetry axis to provide a higher yield of cross-linkages in the presence of linkers, compared to control samples where the assemblies of nanotubes were constructed from the I419CRubisCO solution under the same cross-linking conditions. Reactivities of the Cys residues of I419CRubisCO in crystals and in solution were evaluated using Ellman's reagent (Table 1).^57^ Construction of tubes from a protein solution results in a significantly lower yield of cross-linkages, compared to the nanotubes constructed from protein crystals. The efficiencies of formation of tubes from protein in solution and from protein crystals indicate the same trend observed upon oxidation by 1 mM H_2_O_2_, and co-oxidation with DTT and ED. These results are in agreement with the TEM results. Notably, the protein crystals provide efficient cross-linking for the formation of disulfide bonds which enhances yield *vs.* controls in solution by approximately 20% with H_2_O_2_, 32% with DTT linkers and 27% with ED linkers.

**Table 1:** Yield of cross-linkages calculated from Ellman's assay

Condition	Yield in crystal^*a*^ (%)	Yield in solution^*b*^ (%)
H_2_O_2_	53 ± 7	33 ± 5
H_2_O_2_ with DTT	82 ± 0	50 ± 5
H_2_O_2_ with ED	86 ± 5	59 ± 3

^*a*^Conditions: 1.4 M (NH_4_)_2_SO_4_ and 10 mM MgCl_2_.

^*b*^Conditions: 10 mM MgCl_2_ in 100 mM CHES–NaOH (pH 9.0) at 20 °C for 24 h.

## Discussion

Previous studies demonstrated that a bis-thiol crosslinker would be useful for the construction of supramolecular nanotubes *via* disulfide bonds.^44^ However, the disulfide bonds were formed as a result of auto-oxidation in a buffer solution.^44^ Because the protein molecules freely diffuse in solution, it has remained challenging to regulate the direction of cysteine residues for cross-linking as desired from the initial design. Although it was found that the nanotubes can be elongated to hundreds of nanometers using DTT, the cross-linking sites could not be completely controlled because the intermolecular disulfide bonds were formed randomly with head–head, head–tail, or a mixture thereof.^44^ Furthermore, the numbers of nanotubes were also restricted, when formed in solution, as indicated by the co-existence of the single layers of the non-cross-linked protein.^44^ In our study, protein crystals offer a consistent environment that induces only the formation of face-to-face intermolecular disulfide bonds between the linkers and the Cys419 sites. The efficiency of cross-linking was further enhanced as indicated by elongated nanotubes in TEM images. Thus, the crystal system described herein makes it easy to control the precise protein–protein interactions of interest to stabilize the assembly structure. This cannot be achieved from cross-linking of proteins in solution.

The protein nanotubes from Hcp1 (hemolysin-coregulated protein) were designed from insights obtained using the Hcp1 protein crystal structure.^46^ The layer of nanotubes in the crystalline lattice indicated that the tubular structures could be achieved by the formation of intermolecular disulfide bonds between engineered Cys residues.^46^ Although the nanotubes were demonstrated to form in the buffer solution containing the chemicals for crystallization but milder than the crystallization environment, tube elongation could not be adequately achieved because a high fraction of stacking numbers was identified from 3–6 subunits.^46^ Our current study indicates that utilization of the real 3D crystal could improve the efficiency of cross-linking to promote the elongation of nanotubes into 8–10 stacked layers using DTT and 11–13 stacked layers using ED. These nanotubes are approximately twice as long as the nanotubes obtained in the previous study. It is expected that performing cross-linking reactions within protein crystals will provide a useful advance in extending the size of supramolecular assemblies.

The formation and elongation of protein nanotubes were found to depend on the positions of cysteine residues and C_α_–C_α_ distances between cysteine residues forming cross-linkages. In the present study, the cysteine substitution position was selected in the crystalline matrix at a site where the intermolecular distances between the introduced cysteines would be close to each other. It was found, however, that the Cys419 residues could not form the disulfide bonds efficiently even upon reaction with H_2_O_2_ because the 6 Å C_α_–C_α_ distance between Cys419 residues is apparently too far for the formation of a disulfide bond in the present case. Elongation of nanotubes was achieved by co-oxidation of the I419CRubisCO crystal with cross-linkers (ED and DTT). These reagents access the cross-linking sites and form two pairs of disulfide bonds between their two thiols and two interface Cys residues. Because crystallization reduces the dynamic motion of the protein molecules, the distance between the cross-linked cysteine sites was fixed. Thus, the flexibility and accessibility of linkers are important parameters for the formation of extended nanotubes within the defined crystalline environment that could not be achieved without the presence of linkers. Utilization of cross-linkers enables the formation of intermolecular disulfide bonds at restricted distance sites and enhances the co-oxidation process for the construction of supramolecular assemblies. This strategy can be further applied to different types of protein crystals for the design of sophisticated supramolecular assemblies such as tubes and sheets (Fig. S3†).

RubisCO retains the enzymatic activity in the nanotube structure because the active site is located on the external surface of the tube. Furthermore, the function of the tube was evaluated by the accumulation of Rhodamine B (RB). When RB was reacted with the tube, the dye was immobilized within the tube with an emission peak at 575 nm (Fig. S4†). The peak was slightly shifted to a longer wavelength compared to that of the decamer and free RB (572 nm). It was expected that the shift was caused by π–π interactions of RB with the aromatic groups in the tube.^58^,^59^ The tube structure could provide a unique environment to accumulate external molecules. Thus, the RubisCO nanotubes have the potential for catalysis, and delivery of molecules. We are conducting further research on the design of RubisCO nanotubes for the development of nanobiomaterials.

## Conclusion

In summary, we demonstrated the construction of protein nanotubular structures by engineering of a protein crystal. By crystallization, we could control the protein–protein interaction and the orientation of the engineered cysteine residues at the interface within the crystalline environment. Cross-linking of the introduced cysteine *via* oxidation within the 1D tube assemblies of the crystal played a vital role in retaining the tubular structure after dissolution of the crystal in solution. Furthermore, the elongation of nanotubes could be controlled by co-oxidation of the protein crystal with suitable cross-linkers triggered by the addition of hydrogen peroxide. The nanotubes were stabilized by the intermolecular disulfide bonds between one molecule of cross-linkers and two molecules of adjacent cysteine residues at the interfaces within the crystal. This is considered as a promising strategy for the construction of supramolecular assemblies with sophisticated structures from other protein crystals or for the design of different types of assembled structures from one type of protein with different crystal lattices.

## Conflicts of interest

There are no conflicts to declare.

## Supplementary Material

Supplementary information
