# Supplementary material for: Construction of supramolecular nanotubes from protein crystals
Source: Chem Sci. 2018 Oct 30;10(4):1046–51. doi: 10.1039/c8sc04167a (PMC6346403; doi:10.1039/c8sc04167a)
Supplement: Supplementary file 1 [file SC-010-C8SC04167A-s001.pdf]

Electronic Supplementary Material (ESI) for Chemical Science.
This journal is © The Royal Society of Chemistry 2018

Supporting Information

Construction of Supramolecular Nanotubes from Protein Crystals

Tien Khanh Nguyen, Hashiru Negishi, Satoshi Abe, and Takafumi Ueno*

School of Life Science and Technology, Tokyo Institute of Technology, Nagatsuta-cho, Midori-ku, Yokohama 226-8501, Japan.

Email: tueno@bio.titech.ac.jp

General Information

Materials and Methods

Materials. All chemicals and enzymes were purchased with analytical grade from Sigma – Aldrich, Wako, TCI, Nacalai Tesque, Nanoprobes and used without further purification. The *E. coli* competent cells, including The One Shot® TOP10 for plasmid construction and BL21(DE3) for protein expression were from InvitrogenTM (Life Technologies) and Novagen (EMD Chemicals Inc.), respectively. Primers for site – directed were obtained from Gene Design Inc. PCR was implemented using QuikChange® Site–Directed Mutagenesis Kit (Stratagene).

Construction of recombinant plasmid. Mutant plasmid of RubisCO variant (I419C) constructed in *E. coli* for gene expression was performed by PCR with pET21b (+) plasmid (10 pg/μL in 10 mM Tris-HCl pH 8.0 containing 1 mM EDTA, Novagen) harboring the native gene of *Thermococcus kodakaraensis* KOD1 RubisCO as a template. The primers for the mutant was described through Table S1. Presence of mutation was confirmed by DNA sequencing analysis and then transformed into *E. coli* BL21(DE3) for protein expression.

Protein expression and purification. Expression and purification of protein were performed as previously described with any modifications.[1] The *E. coli* BL21(DE3) cells were grown in *Luria broth* (LB) medium containing 50 mg/mL ampicillin at 37°C. The protein expression was induced for 4 h until optical density (OD) reached of 0.5 at 660 nm by addition of 0.1 mM isopropyl-D-thiogalactopyranoside (IPTG) and then the cells were harvested by centrifugation (7500 rpm, 5 min, 4°C). The protein was extracted by resuspending the harvested cells in 20 mM Tris-HCl (pH 8.0), 10 mM $MgCl_2$ and sonication (on ice), followed by centrifugation (17500 rpm, 30 min, 4°C). The supernatant was heated for 30 min at 85°C and the precipitates were eliminated by centrifugation (17500 rpm, 30 min, 4°C). The heat-treated samples were purified with HiTrap Q HP 5 x 1 mL column (GE Healthcare, Little Chalfont, UK) eluting with NaCl gradient (0 – 1.0 M) in 20 mM Tris-HCl (pH 8.0), 10 mM $MgCl_2$, following by further purification with AKTA Sephadex G200 column (GE Healthcare, Little Chalfont, UK) equilibrating with 50 mM Sodium Phosphate (pH 7.0) and 0.15 M NaCl. Prior to crystallization, the purified RubisCO was subjected

to Resource Q column 1 mL (GE Healthcare, Little Chalfont, UK) for further purification and dialyzed against 100 mM Bicine-KOH (pH 8.3), 10 mM $MgCl_2$ for 12 hours at room temperature.

Protein crystallization. Crystals were grown using hanging-drop vapor diffusion method at 20°C.[2] Crystals of I419CRubisCO were observed within 3 days from a protein solution of 30 mg/mL protein solution, 1.4 M $(NH_4)_2SO_4$, 10 mM $MgCl_2$ in 100 mM CHES-NaOH (pH 9.0).

Confirmation of crystal lattices by TEM. Crystals of I419CRubisCO were cross-linked with 1% glutaraldehyde for 24 h at 20°C. Subsequently, crystals were washed with appropriate buffer (similarly to mother solution of crystallization with ammonium sulfate in CHES-NaOH buffer (pH 9.0)) and cracked into small pieces. Small pieces of crystals were subjected into the grids and stained similarly to procedure of preparation for TEM described below.

Construction of Protein Nanaotubes from the mutant I419CRubisCO crystals.

Reaction of I419CRubisCO crystals with H_2O_2. Crystals of I419CRubisCO was added to a solution of 1 mM H_2O_2 in CHES-NaOH buffer (pH 9.0), 1.4 M $(NH_4)_2SO_4$. All the cross-linked crystals were harvested and transferred into the buffer solution of 100 mM Glycine-NaOH (pH 9.0) for 12 h at 20°C for dissolution without exogenous disruption. Control experiments were carried out in protein solution of 5 mg/mL I419CRubisCO, 1 mM H_2O_2 in 100 mM CHES-NaOH (pH 9.0) for 24 h, 20°C.

Co-oxidation of I419CRubisCO with cross-linkers. Crystals of I419CRubiCO were added to the solution containing 25 mM cross-linker (Dithiothreitol DTT or 1,2-Ethanedithiol ED) for 8 h, and then incubated in 1 mM H_2O_2 for 16 h at 20°C. Finally, all the cross-linked crystals were harvested and transferred into the buffer solution of 100 mM Glycine-NaOH (pH 9.0) for 12 h at 20°C for dissolution without exogenous disruption. Control experiments were carried out in protein solution of 5 mg/mL I419CRubisCO with the same procedure for 25 mM cross-linker in 8 h and then 1 mM H_2O_2 in 16 h.

Accumulation of dye molecules in the tubular assemblies. Crystals of I419CRubisCO were soaked with 1 mM Rhodamine B in CHES-NaOH buffer (pH 9.0), 1.4 M $(NH_4)_2SO_4$ for 3 days at

20°C. Subsequently, all crystals were washed three times with the same buffer to eliminate unbound dyes, cross-linked, dissolved, and recorded with fluorescence spectra. For cross-linking, crystals were soaked in the solution of 25 mM ED (20°C, 8 h) and 1 mM H_2O_2 (20°C, 16 h). All the cross-linked crystals were harvested and transferred into the buffer solution of 100 mM Glycine-NaOH (pH 9.0) for 12 h at 20°C for dissolution without exogenous disruption. Luminescence Spectroscopy of protein nanotubes with the immobilized functional dyes was recorded on a F-7000 fluorescence spectroscopy with the emission wavelength of 553 nm.

Transmission Electron Microscopy (TEM). The images were implemented using a 120 kV JEOL 1400-Plus (JEOL, Tokyo). The RubisCO samples used were either the non-oxidized form or oxidized forms (Wild-type, I419CRubisCO, and nanotubes). Prior to visualization, all samples were prepared with dilution up to final concentration of 3.5 mg/mL protein. 5 µL of sample was loaded to a carbon-coated copper grid for 1 minute; then the excess liquid on grid was discarded and the grid was washed with 5 µL of distilled water. The sample was negatively stained twice for 1 minute with 5 µL, 1 % Methylamine Tungstate (Nanoprobes, CAS No. 55979-60-7). Samples were imaged on JEOL1400-Plus electron microscopes at 80 kV.

Evaluation of enzymatic activity of RubisCO. The carboxylase activity of RubisCO was determined as described previously.[3] The 10 µL, 0.2 mg/mL of I419CRubisCO or RubisCO nanotube were activated in 100 mM Bicine-NaOH (pH 8.3), 10 mM $MgCl_2$, 1 M $NaHCO_3$ for 16 h at 25°C prior to the enzymatic assay. 10 µL of 2 mM NADH (CAS No. 104809-32-7), 50 µL of 10 mM ATP (CAS No. 34369-07-8) and 10 mM reduced glutathione (CAS No. 70-18-8), 10 µL of coupling enzymes were added to 20 µL of activated RubisCO at 25°C. The mixture of coupling enzymes contains 563 units/mL 3-phosphoglycerate kinase, 125 units/mL glyceraldehyde-3-phosphate dehydrogenase, 260 units/mL triose phosphate isomerase and 22.5 units/mL glycerol-3-phosphate dehydrogenase. The enzymatic reaction was initiated by addition of 10 µL, 100 mM Ribulose-1,5-bisphosphate (RuBP, CAS No. 24218-00-6) to 100 µL reaction solution at 25°C. The enzyme activity was calculated from the slope of the UV absorbance change at 340 nm. The experiments were conducted three times.

Determination of Free Thiol for calculation of cross-linking yield. The free thiol cysteine concentration, corresponding to the non-oxidized cysteine group was determined by Ellman's Assay using L-Cysteine as standard.[4] 5 μL of sample was added to 190 μL solution of Ellman's Reagent (5,5'-Dithio-bis-(2-nitrobenzoic acid) (CAS No. 69-78-3), and 5 μL of distilled water. Subsequently, the reaction was placed for 5 min at 20°C. The free thiol concentration was calculated from measuring absorbance at 412 nm and using Cysteine standard curve. The yield (%) of covalent cross-linking was defined as below: % Yield = (Free Thiol/ Total Thiol) x 100

Statistical Data Analysis. Statistical data analyses were carried out using a Student's t-test method. Values of $P < 0.05$ were considered statistically significant.

Figure and Table

Table S1. Primers used for site-directed mutagenesis

Mutant	Template Plasmid	Sequence
I419C	pET/rbc$_{TK}$	5'- CATAATGCAGGGATGCCCGCTCGACGAGTACG -3'<br>5'- CGTACTCGTCGAGCGGGCATCCCTGCATTATG -3'

Table S2. Enzymatic activity of RubisCO

Mutant	Specific activity (μmole/min.mg)
I419CRubisCO	0.143 ± 0.015
RubisCO nanotube	0.127 ± 0.007

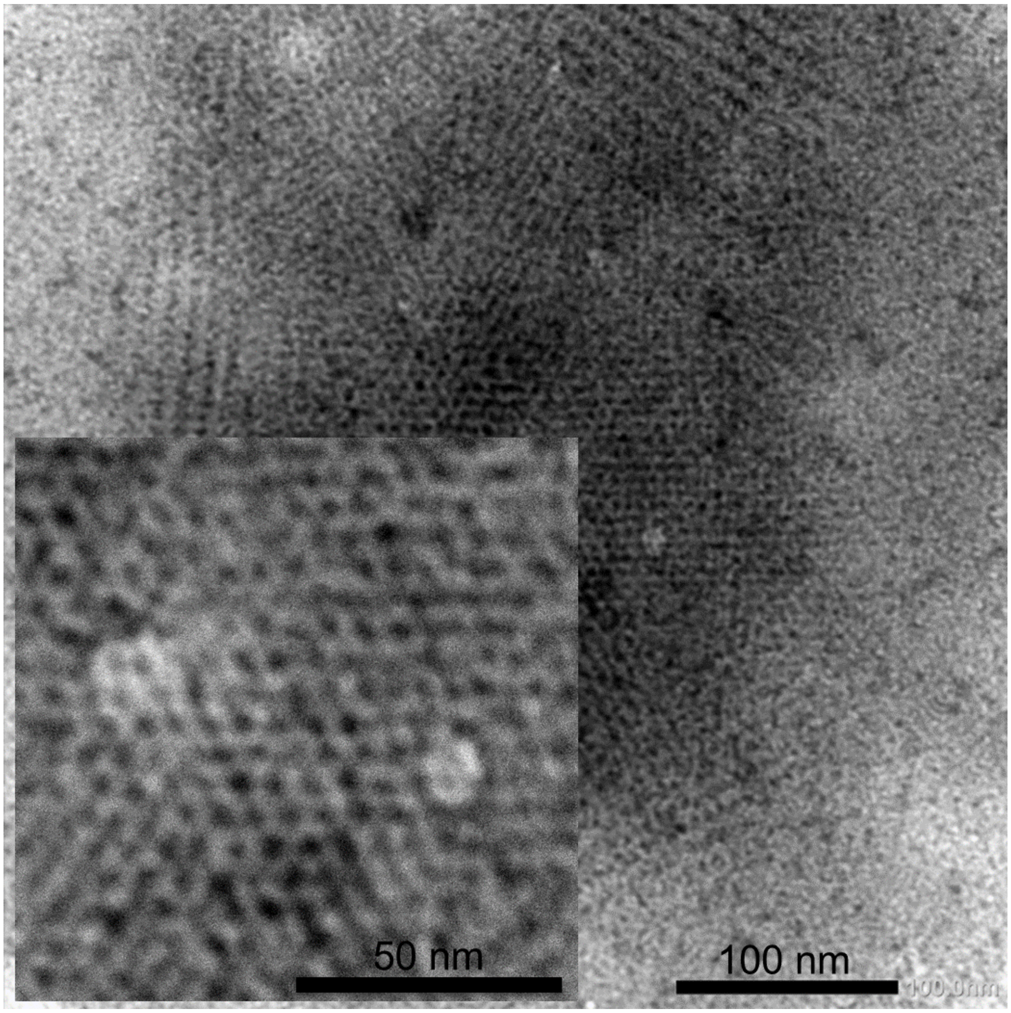

Fig. S1 TEM images of lattice structure of I419CRubisCO crystal confirmed by TEM after random cross-linking with 1% glutaraldehyde. Crystals were randomly cross-linked with 1% glutaraldehyde (24 h, 20°C), washed with mother solution of crystallization, then cracked into small pieces, and loaded into grids for observation by TEM.

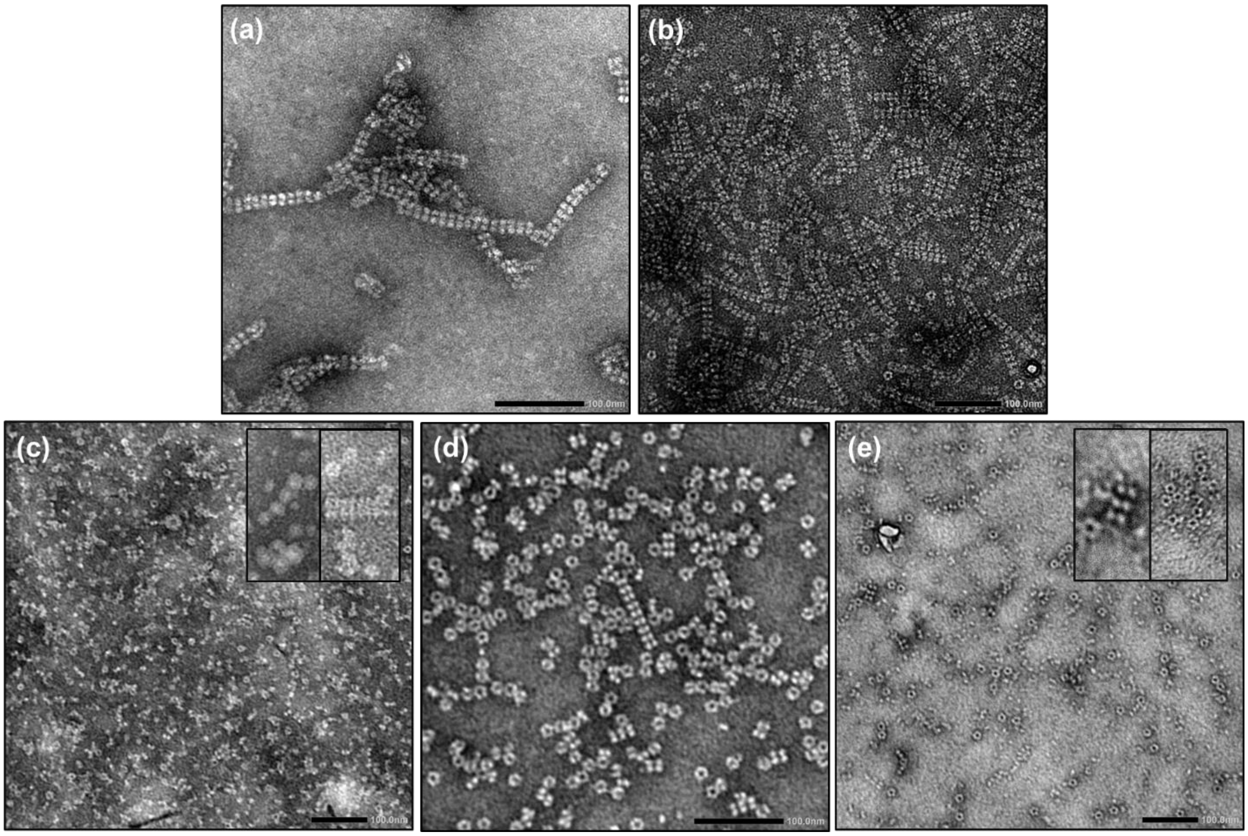

Fig. S2 TEM figures of experiments using variant I419CRubisCO for construction of nanotubes. (a) Tube formation with employment of I419CRubisCO crystals exposed into a solution of 25 mM ED and 1 mM H_2O_2. (b) Tube formation with employment of I419CRubisCO crystals exposed into a solution of 25 mM DTT and 1 mM H_2O_2. (c) Oxidation of I419CRubisCO solution triggered by 1 mM H_2O_2 showed a low stacking number of 4 and random aggregation. (d) 25 mM ED and 1 mM H_2O_2 triggered the formation of disulfide bonds into short tubes with 9 stacking numbers and random aggregation when I419CRubisCO solution was accommodated. (e) A short tube with 3 stacking numbers and random cross-linking were observed when I419CRubisCO solution was cross-linked with 25 mM DTT and 1 mM H_2O_2. Scale bar: 100 nm.

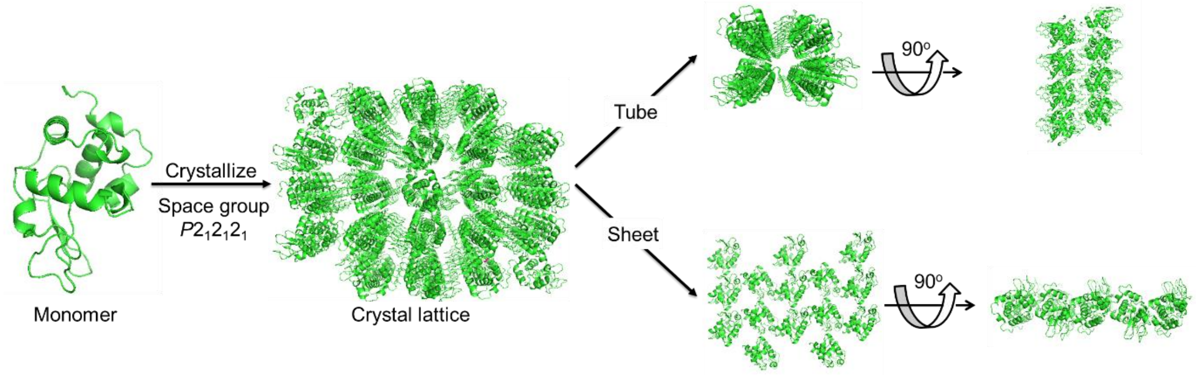

Fig. S3 Examples of construction of supramolecular protein assemblies via cross-linking of protein crystal. Crystallization of hen egg-white lysozyme (HEWL) into orthorhombic morphology with the space group of $P2_12_12_1$ (pdb id: 1bgi).[5] The crystal lattice indicates the assembly structures of tube, and sheet.

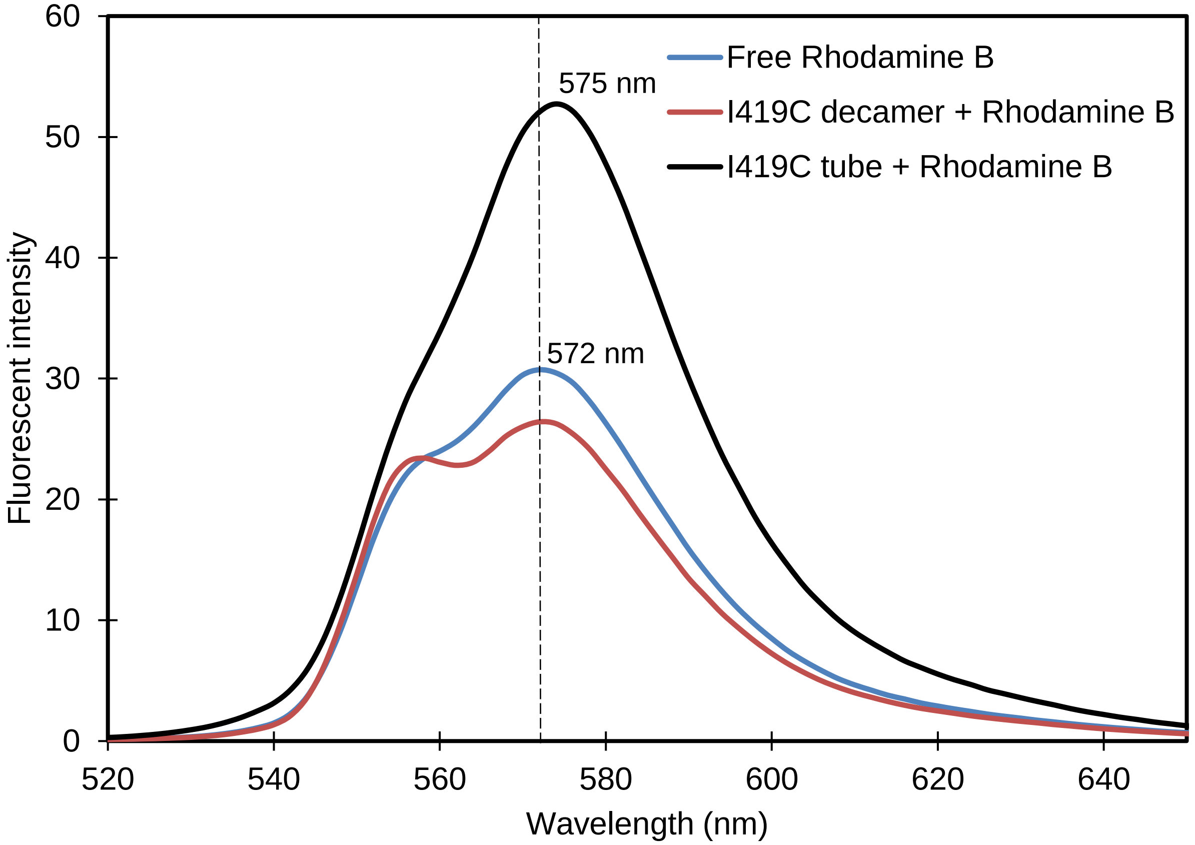

Fig. S4 Fluorescent spectra of protein assembly after immobilization of functional dye Rhodamine B into protein nanotubes. This spectrum shows the potential of RubisCO nanotubes as a template for immobilization of functional materials, indicated by the strong intensity near the emission wavelength of 572 nm of free Rhodamine B with a slight shift to 575 nm. Excitation wavelength: 553 nm.

References

(1) Ezaki, S.; Maeda, N.; Kishimoto, T.; Atomi, H.; Imanaka, T. *J. Biol. Chem.* **1999**, *274*, 5078.

(2) Maeda, N.; Kanai, T.; Atomi, H.; Imanaka, T. *J. Biol. Chem.* **2002**, *277*, 31656.

(3) Fujihashi, M.; Nishitani, Y.; Kiriyama, T.; Aono, R.; Sato, T.; Takai, T.; Tagashira, K.; Fukuda, W.; Atomi, H.; Imanaka, T. *Proteins: Struct. Funct. Bioinform.* **2016**, *84*, 1339.

(4) Ellman, G. L. *Arch. Biochem. Biophys.* **1959**, *82*, 70.

(5) Oki, H.; Matsuura, Y.; Komatsu, H.; Chernov, A. A. *Acta Crystallogr. D* **1999**, *55*, 114.
